# The influence of perilipin 5 deficiency on gut microbiome profiles in murine metabolic dysfunction-associated fatty liver disease (MAFLD) and MAFLD-hepatocellular carcinoma

**DOI:** 10.3389/fcimb.2024.1443654

**Published:** 2024-10-14

**Authors:** Marinela Krizanac, Paula Štancl, Paola Berenice Mass-Sanchez, Rosa Karlić, Diana Moeckel, Twan Lammers, Anastasia Asimakopoulos, Ralf Weiskirchen

**Affiliations:** ^1^ Institute of Molecular Pathobiochemistry, Experimental Gene Therapy and Clinical Chemistry (IFMPEGKC), RWTH University Hospital Aachen, Aachen, Germany; ^2^ Bioinformatics Group, Division of Molecular Biology, Department of Biology, Faculty of Science, University of Zagreb, Zagreb, Croatia; ^3^ Institute for Experimental Molecular Imaging, RWTH Aachen, Aachen, Germany

**Keywords:** microbiome, fatty liver disease, animal models, hepatocellular carcinoma, metabolic syndrome, metabolic dysfunction, microbial diversity

## Abstract

**Introduction:**

Metabolic dysfunction-associated fatty liver disease (MAFLD) has emerged as the leading cause of hepatocellular carcinoma (HCC) worldwide. Over the years, Perilipin 5 (PLIN5) has been recognized as a key regulator of both MAFLD and HCC development. In our previous studies we demonstrated that deficiency in *Plin5* reduces the severity of MAFLD and HCC in mice. Interestingly, it has been established that patients with MAFLD and HCC exhibit various changes in their gut microbiome profiles. The gut microbiome itself has been shown to play a role in modulating carcinogenesis and the immune response against cancer.

**Methods:**

Therefore, we conducted a study to investigate the alterations in fecal microbiome composition in wild type (WT) and *Plin5*-deficient (*Plin5* ^-/-^) mice models of MAFLD and MAFLD-induced HCC (MAFLD-HCC). We utilized 16S rRNA gene sequencing analysis to profile the composition of gut bacteria in fecal samples.

**Results:**

Notably, we discovered that the absence of *Plin5* alone is already associated with changes in gut microbiota composition. Moreover, feeding the mice a Western diet (WD) resulted in additional microbial alterations. Interestingly, *Plin5* ^-/-^ animals exhibited an enrichment of the beneficial taxa *Lactobacillus* in both animal models.

**Discussion:**

Our findings identify *Plin5* as a major regulator of gut microbiota during the development of MAFLD and MAFLD-HCC.

## 1 Introduction

Within the past decade, there has been increasing evidence of a correlation between the composition of gut microbes and the development of liver diseases (Hrncir et al., 2021). This interaction, in which gut bacteria regulate adaptive immunity, inflammation, metabolism, nutrient absorption, and liver diseases, is known as the gut-liver axis (Albillos et al., 2020). Mechanistically, there are several ways in which the microbiota can regulate and contribute to liver disease. Firstly, pathogenic gut bacteria can overgrow, affect gut permeability, and release lipopolysaccharides, leading to systemic inflammation. Other ways include the release of bacterial metabolites (such as trimethylamine *N*­oxide, lactate, choline or ethanol) and the conversion of bile acids into toxic substances (Zhou et al., 2022; Collins et al., 2023). Bacteria have enzymes that convert primary bile acids into secondary bile acids, allowing them to modulate the primary bile acid pool and activate intestinal farnesoid X receptor signaling, a pathway that promotes metabolic dysfunction-associated fatty liver disease (MAFLD) (Collins et al., 2023).

Interestingly, while animal studies have confirmed the causal roles of gut bacteria through coprophagy and fecal transfer studies, human studies have just started to detect microbial signatures that allow discrimination between patients with MAFLD, cirrhosis and healthy individuals (Aron-Wisnewsky et al., 2020). Across the literature, significant differences or even opposing results can be found in the composition of microbial taxa that are relevant in MAFLD, metabolic dysfunction-associated steatohepatitis (MASH) and HCC (Gupta et al., 2022; Albhaisi and Bajaj, 2021). However, some common trends in the composition of the microbiome have been observed at the phylum and genus levels (Aron-Wisnewsky et al., 2020). Generally, an increase in the abundance of *Firmicutes* and *Proteobacteria*, accompanied by a decrease in *Bacteroides* is often observed at the phylum level in MAFLD. Additionally, at the genus level, there is an increase in *Akkermansia*, *Escherichia*, *Ruminococcus* and *Shigella*, while levels of taxa that are often considered beneficial such as *Alistipes*, *Eubacterium* and *Lactobacillus* are reduced. These alterations are often accompanied by a decrease in microbial diversity (Astbury et al., 2020; Jiang et al., 2023).

Perilipin 5 (PLIN5), a member of the perilipin family mostly found in lipid droplets (LD) has recently been studied in the context of MAFLD, MASH and their progression towards HCC. As we and others have previously reported, *Plin5* deficiency can alter the progression of MAFLD by reducing inflammasome formation, hepatic injury, and steatosis (Asimakopoulou et al., 2020; Ma et al., 2021). Furthermore, we have suggested that PLIN5 is a critical factor that drives formation of HCC (Asimakopoulou et al., 2019). Considering that PLIN5 is a crucial regulator of MAFLD-induced HCC (Krizanac et al., 2023) and that its depletion markedly reduces the progress of MAFLD and attenuates MAFLD-HCC formation in two animal models (Mass-Sanchez et al., 2024), the question has arisen about whether these findings are also characterized by specific changes at the gut microbiome level. To investigate this, fecal samples were collected from WT and *Plin5*-deficient mice of MAFLD and MAFLD-HCC models prior to sacrifice, and 16S rRNA gene sequencing analysis was performed to reveal any possible altered microbial diversity.

## 2 Materials and methods

### 2.1 Animals

The mice strain targeted disrupted for the *Plin5* gene was generated by inseminating female mice with *Plin5* ^-/-^ sperm obtained from the Jackson Laboratory, Bar Harbor, ME, USA using a protocol previously decribed (Kuramoto et al., 2012; Asimakopoulou et al., 2020). Wild type (WT) and *Plin5^-/-^* mice were bred on a C57BL/6J background and housed at the Institute for Laboratory Animal Science and Experimental Surgery at RWTH-Aachen University, with a constant temperature of 20°C, relative humidity of 50%, and a 12-hr on/off light cycle. Standard cages housed up to four animals of the same genotype and diet. All animals in this study received proper care, and all animal protocols followed the guidelines for animal care approved by German legislation on the protection of animals. The protocols were also approved by the responsible authority of the state of North Rhine-Westphalia (LANUV, Recklinghausen, Germany) under permit no.: 81-02.04.2019.A366.

### 2.2 Animal model

The two animal models of MAFLD and MAFLD-induced HCC (MAFLD-HCC) and sources of animals used in the study have been previously described (Asimakopoulou et al., 2020; Mass-Sanchez et al., 2024). In brief, the MAFLD-HCC model involved applying 7,12-dimethylbenz(a)anthracene (DMBA) once to the dorsal neck surface of 4-5 day old mice, followed by feeding them a Western diet (WD) (D17010102, Research Diets, Inc., New Brunswick, NJ, USA) for 30 weeks after weaning. In the MAFLD model, the DMBA was replaced with acetone (AC). The WD consisted of a high-fat diet (D17010102, Research Diets, Inc., New Brunswick, NJ, USA) and water *ad libitum* supplemented with glucose/fructose (42 g/L; 55% fructose and 45% glucose), which was renewed every two weeks. Control animals were given a normal diet (ND) (V1534, ssniff Spezialdiäten GmbH, Soest, Germany) and drinking water without the sugar supplement. Animal body weight and food intake were monitored weekly.

### 2.3 Micro-computed tomography

Micro-computed tomography (µCT) and fat scans in mice were essentially performed as previously described (Kroh et al., 2023).

### 2.4 Gut microbiome analysis

All animals analyzed were kept in the same room and on the same cage rack to minimize the microbial variability caused by external environmental factors. The fecal samples (n=4 per group) for microbiome analysis were collected directly into Eppendorf tubes from freshly defecated animals to avoid environmental contamination prior to sacrifice. Fecal samples were then snap frozen in liquid nitrogen and stored at -80°C until further processing. Subsequent steps, such as DNA extraction and sequencing, were performed by professionals at the Institute of Medical Microbiology of RWTH University Hospital in Aachen.

### 2.5 DNA extraction

Microbial DNA was extracted using a slightly modified version of a previously published protocol (Godon et al., 1997). In brief, 600 µL of fecal sample in DNA stabilizer was transferred to autoclaved bead-beater tubes containing 500 mg ± 10 mg of triple-pure 0.1 mm Zirconia/Silica beads (#55D1132-01TP, Biozym Scientific GmbH, Hessisch Oldendorf, Germany). Then, 250 µL 4 M guanidine thiocyanate in 0.1 M Tris pH 7.5 and 500 µL of 5% *N*-laurolylsarcosine in phosphate-buffered saline were added and vortexed. The samples were incubated at 70°C for 60 minutes on a shaker with a low shaking frequency (700 rpm). Bacterial cell walls were disrupted in the Fast Prep-24™ 5G Bead Beating Grinder and Lysis System (MP Biomedicals, Schwerte, Germany) using a bead-beating program consisting of 3 repetitions for 40 seconds at 6.6 m/s. The cooling adapter of the bead-beater was refilled with dry ice between each round. Next, 15 mg of poly(vinylpolypyrrolidone) was added to the samples and the samples were vortexed. The samples were centrifuged at 15,000 x g and 4°C for 3 minutes. The clear supernatants were transferred to new 2 mL Eppendorf tubes and centrifuged again at 15,000 x g and 4°C for 3 minutes. 500 μL of each clear supernatant was transferred into a new 2 mL tube, followed by the addition of 5 μL of RNase solution (10 mg/ml). After incubation at 37°C while shaking (700 rpm) for 20-30 minutes, genomic DNA (gDNA) was extracted using the NucleoSpin^®^ gDNA Clean-up protocol (#740230.50, Machery-Nagel, Düren, Germany) according to the manufacturer’s instructions.

### 2.6 Sequencing

The 16S rRNA gene amplicon profiling was conducted by the Functional Microbiome Research Group at the Institute of Medical Microbiology, RWTH University Hospital Aachen, Aachen, Germany. To begin, library preparation was performed using a pipetting robot (Biomek4000 Beckman Coulter, Krefeld, Germany) for standardized processing. This was done following a previously published protocol (Lagkouvardos et al., 2016). The V3/V4 region of the 16S rRNA genes was amplified in two rounds of PCR in duplicates (15 cycles + 10 cycles) using bacteria-specific primers 341F/785R. Amplicon purification was carried out using the AMPure XP system (Beckman), followed by paired-end modus (PE275) sequencing using a MiSeq system (Illumina, Inc., San Diego, CA, USA) as per the manufacturer’s instructions. The final DNA concentration was adjusted to 10 pM with a 15% (v/v) PhiX standard library.

### 2.7 Microbiome data analysis

Raw read files were processed with UPARSE based analysis (Edgar, 2013) using the IMNGS pipeline (Lagkouvardos et al., 2016). Sequences were de-multiplexed and trimmed to first base with a quality score <3 and then paired. Sequences with <250 and >500 nucleotides and paired reads with an expected rate of 0.002 were excluded. Reads were trimmed by 10 nucleotides on both sides. Pairing, quality filtering and OTU clustering (97% identity) was done by USEARCH 11.0 (Edgar, 2010). Removal of non 16S sequences was done with SortMeRNA v4.2 with SILVA release 138 as a reference (Kopylova et al., 2012). Clustering of Operational taxonomic units (OTUs) was done at 97% sequence similarity. Only OTUs with relative abundance over 0.25% across all samples were kept. **Supplementary Table S1** provides an overview of the number of OTUs before and after each IMNGS filtering step. Sequence alignment and taxonomic classification were done by SINA 1.6.1 using the taxonomy of SILVA release 138 (Pruesse et al., 2012). The OTU table from the IMNGS pipeline was used in downstream analyses using the R package Rhea (Lagkouvardos et al., 2016). Rarefaction curves were calculated on OTUs normalized via division by the sum of sequences in a given sample and multiplication by the minimum sum across all samples. Alpha diversity was calculated based on the Shannon index (Shannon, 2001) and observed OTUs using function richness from R package microbial (Guo and Gao, 2021). Beta diversity analysis between groups of diets was calculated using the Bray-Curtis distance. Principal coordinate analysis (PCoA) ordination based on calculated Bray-Curtis distance was used to visualize the dispersion between the groups. A permutational multivariate analysis of variance (PERMANOVA) using the adonis2 function in the package vegan (Oksanen et al., 2020), was applied to test significant clustering between sample groups. The relative abundances at different taxonomical levels were generated in each sample using the tax_glom command in the R package phyloseq (McMurdie and Holmes, 2013) and shown with stacked bar charts. The taxonomic relative abundances in samples were visualized by heatmap from R package pheatmap (Kolde, 2019). To analyse differential bacterial taxa abundance between diets and genotypes, a linear discriminant analysis (LDA) effect size (LEfSe) method with a threshold of logarithmic LDA score 2.0 and FDR < 0.05 (Segata et al., 2011) was used on the OTU table. Functional analysis of microbiomes was conducted with Phylogenetic Investigation of Communities by Reconstruction of Unobserved States (PICRUSt2) (Douglas et al., 2020). PICRUSt2 was used to predict the abundances of functional categories in the Kyoto Encyclopedia of Genes and Genomes (KEGG) (Kanehisa et al., 2011), orthologs (KO), Enzyme Commission numbers (EC numbers) and metabolic pathways from MetaCyc database (Caspi et al., 2014). ALDEx2 (Fernandes et al., 2014) algorithm was applied to calculate the effect size of KOs and conducted significant testing in the comparison between groups. Due to a low number of significant terms after multiple hypothesis testing, we identified KOs and ECs with an uncorrected *p*-value below 0.1 as distinct using Wilcoxon rank sum. Distinct KOs were used for KEGG over-representation analysis (ORA) with R package clusterPofiler (Yu et al., 2012) and significant terms were reported with Benjamin-Hochberg (BH) adjusted *p*-value < 0.05. Moreover, we calculated the mean proportion of distinct KO terms with error bars showing standard deviation (SD) across all biological samples of the selected pathways involved in the prevention of hepatocarcinogenesis from ORA. R package ggplot2 (Wickham, 2016) was used to construct boxplots and bar charts shown in the microbiome analysis part of the research. The significance was compared using Welch’s test, or the Wilcoxon rank sum test with Benjamini-Hochberg correction. Other statistical approaches in bioinformatics analyses have been described above. R version 4.3.3 (R foundation for Statistical Computing).

## 3 Results

### 3.1 The loss of *Plin5* reshapes the gut microbiome of animals fed a normal diet

**Figure 1:**
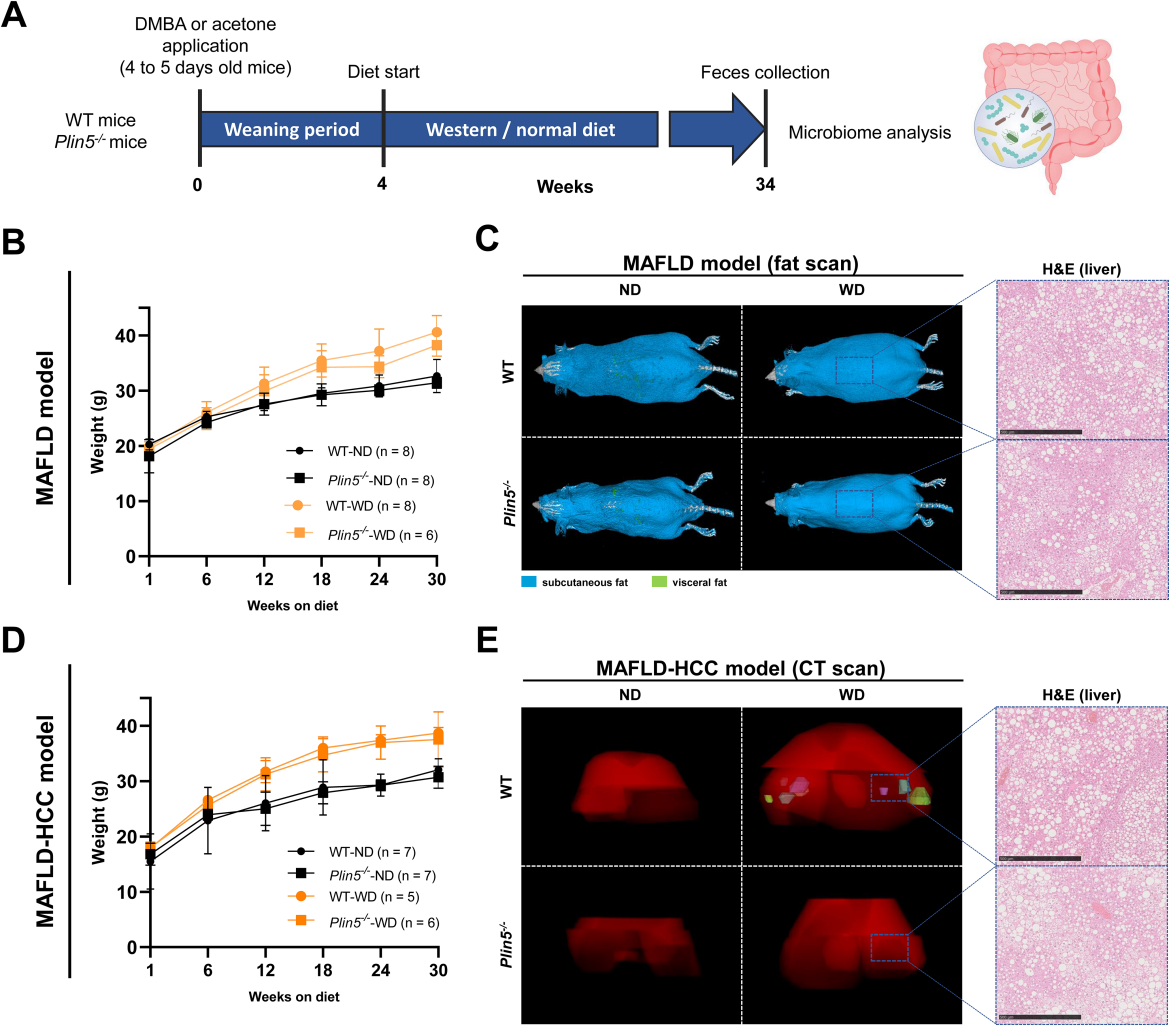
MAFLD and MAFLD-HCC models exhibit characteristic changes for fatty liver disease. **(A)** Schematic representation of the MAFLD and MAFLD-HCC models. The treatment with acetone or DMBA was followed by a 4-week weaning period and normal or Western diet feeding for 30 weeks. Fecal samples were collected immediately prior to sacrifice. **(B)** Weight measurements of animals in the MAFLD model. Number of animals per group: WT-ND (n=8); WT-WD (n=8); *Plin5* ^-/–^ND (n= 8); *Plin5* ^-/–^WD (n= 6) **(C)** Fat scans displaying subcutaneous fat in blue and visceral fat in green. Haematoxylin and eosin (H&E) staining of liver tissues from animals in the MAFLD model. Bars indicate 500 µM. **(D)** Weight measurements of animals in the MAFLD-HCC model. Number of animals per group: WT-ND (n=7); WT-WD (n=5); *Plin5* ^-/–^ND (n=7); *Plin5* ^-/–^WD (n=6) **(E)** Computed tomography (CT) scans displaying the 3D liver in red and tumors in multiple colors (left panels). H&E staining of liver tissue from animals in the MAFLD-HCC model (right panels). Abbreviations used are: wild type (WT); *Plin5*-deficient (*Plin5^-/-^*); normal diet (ND); Western diet (WD).

The schematic representation of the time course for the selected models can be seen in **Figure 1A**. More detailed information regarding the characteristics of these models can be found elsewhere (Mass-Sanchez et al., 2024). When comparing WD-fed animals treated with AC (MAFLD model) to ND-fed animals, it was observed that the former displayed higher weight gain, body fat accumulation and liver fat accumulation (**Figures 1B, C**). Interestingly, the loss of *Plin5* was found to be beneficial in reducing inflammation caused by MAFLD in this particular context. As anticipated, the MAFLD-HCC model in WT exhibited increased weight gain, tumor formation and steatosis in WD-fed animals when compared to their ND-fed counterparts (**Figures 1D, E**). Notably, the absence of hepatic tumor formation was observed in the MAFLD-HCC model when *Plin5* deficiency was present.

**Figure 2:**
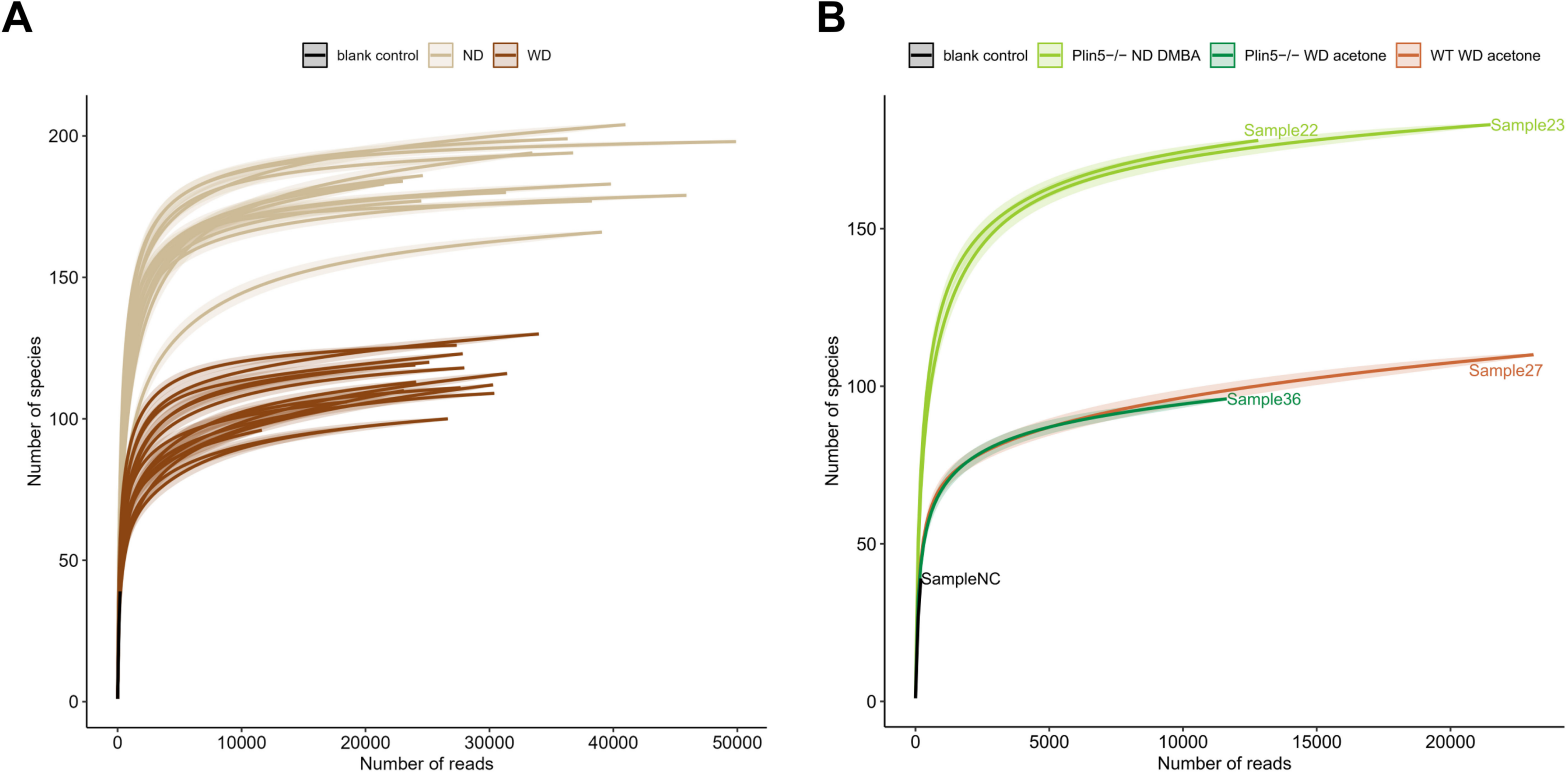
Rarefaction curves demonstrate a satisfactory level of saturation in all analyzed samples. **(A)** Rarefaction curves of all samples utilized in the study, including the negative control, are color-coded by the mouse diet **(B)** The figure highlights cases with the least sampling, colored-coded by mouse genotype and administered treatment.

To analyze whether *Plin5* is implicated in microbiome regulation, fecal samples were collected from animals subjected to the MAFLD and MAFLD-HCC models. 16S rRNA gene sequencing was performed to analyze bacterial composition. Rarefaction curves were generated to detect under-sampled cases. These curves showed that all samples used in the study had a satisfactory level of saturation necessary for the analysis (**Figure 2**).

**Figure 3:**
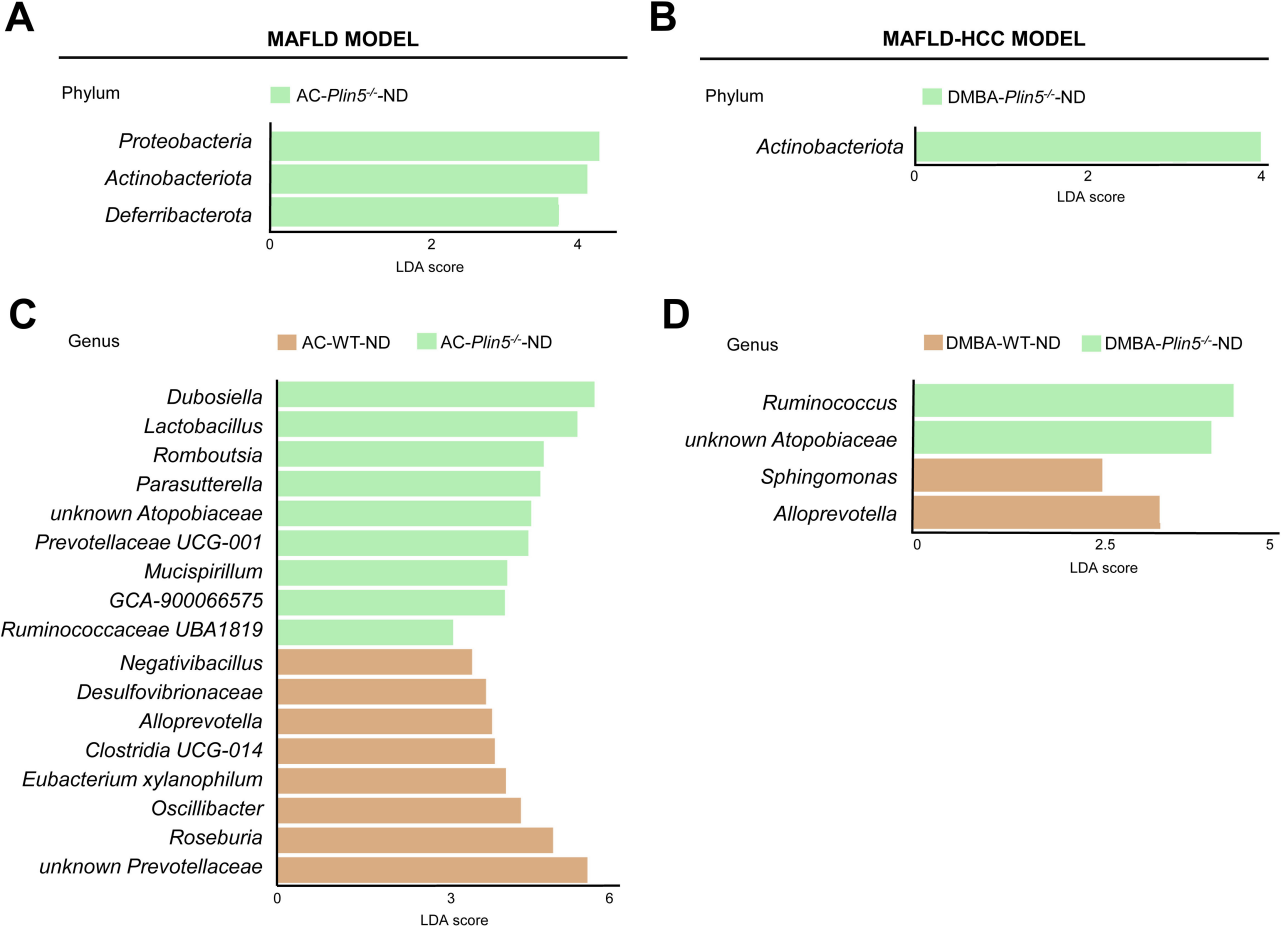
*Plin5* deletion is sufficient to modify the microbial composition of mice fed a normal diet at the phylum and genus levels. **(A–D)** Linear discriminant analysis (LDA) effect size (LEfSe) was used to compare taxa at the phylum and genus levels in fecal samples between *Plin5*-deficient (*Plin5^-/-^)* and wild type (WT) mice in the MAFLD and the MAFLD-HCC models. *Actinobacteriota* are the common taxa upregulated by the loss of *Plin5* in both animal models. At the genus level, the deficiency of *Plin5* led to changes in the abundances of *unknown Atopobiaceae* and *Alloprevotella*. Abbreviations used are: normal diet (ND); Western diet (WD); 7,12-dimethylbenz(a)anthracene (DMBA); acetone (AC).

Fecal 16S rRNA sequencing results of ND-fed *Plin5^-/-^* animals treated with either the vehicle AC or DMBA showed that *Plin5* deletion changed the bacterial composition at both the phylum and genus levels in the MAFLD model when compared to WT control littermates. The LDA effect size (LEfSe) was used to calculate the taxa that discriminated between WT and *Plin5* ^-/-^ mice (**Figures 3A, B**). Specifically, the *Proteobacteria*, *Actinobacteriota* and *Deferribacterota* phyla showed increased abundances in *Plin5^-/-^* mice compared to WT animals. Additionally, there were 17 differences found between the WT and *Plin5^-/-^* groups at the genus level (**Figure 3C**). While WT animals displayed higher levels of *Prevotellaceae*, *Roseburia*, and *Oscillibacter* as the top three upregulated genera, *Plin5* knockout animals were characterized by elevated abundances of *Dubosiella*, *Lactobacillus* and *Romboutsia*.

Similar to the MAFLD model, depletion of *Plin5* also changed the bacterial composition at the phylum level in animals that received DMBA. The relative abundance of *Actinobacteriota* greatly increased in *Plin5^-/-^* animals fed with ND compared to WT controls. Only four bacterial taxa were found to be differentially represented in WT *vs*. *Plin5^-/-^* animals at the genus level. The loss of *Plin5* resulted in an increase in the abundance of *Ruminococcus* and *Atopobiaceae*, and a decrease in *Sphingomonas* and *Alloprevotella* (**Figure 3D**). **Supplementary Table S2** provides a summary of the shared changes in the microbiome between the MAFLD and MAFLD-HCC models caused by *Plin5* depletion.

### 3.2 The Western diet leads to specific changes in the gut microbial composition in both models

**Figure 4:**
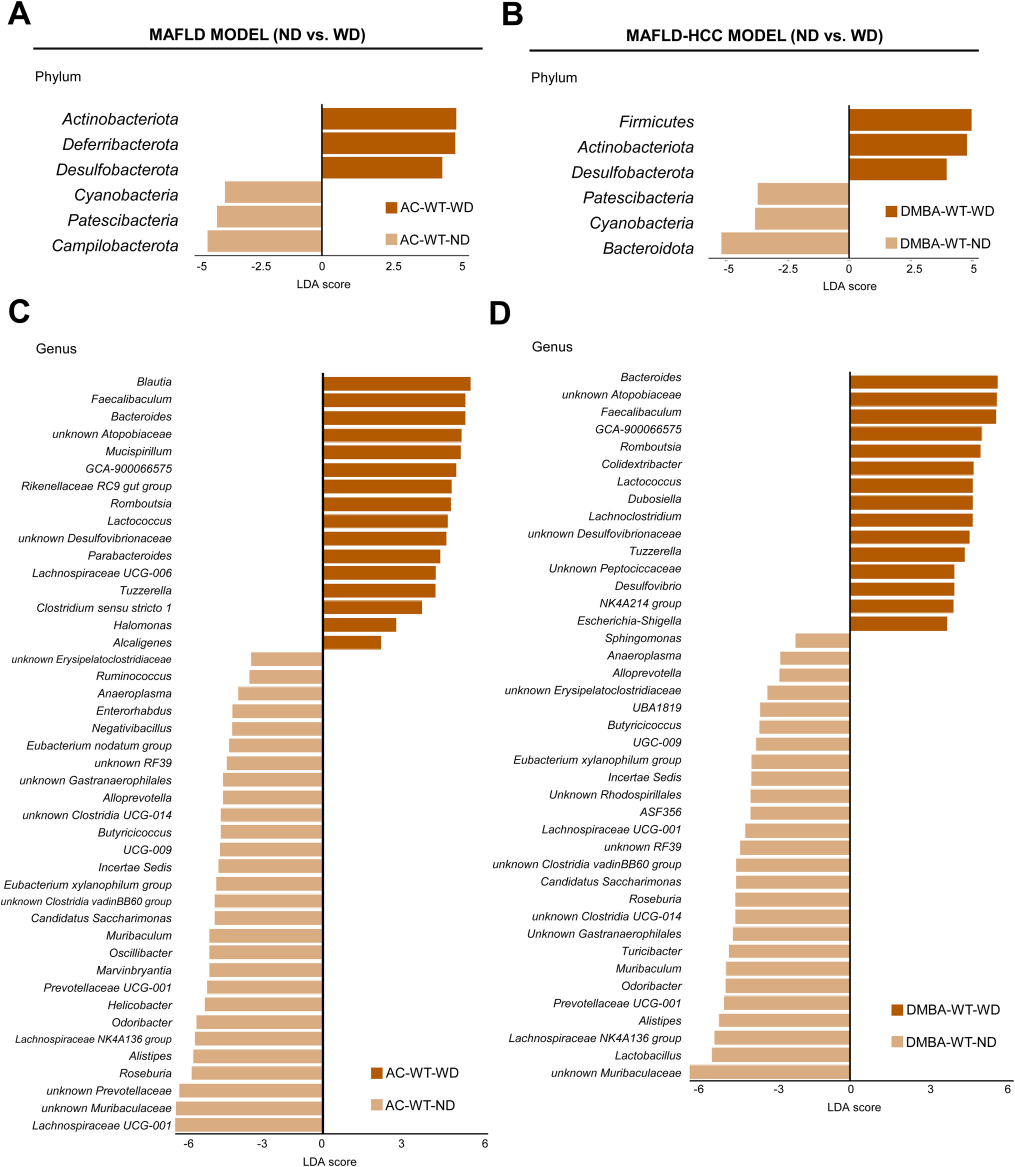
The Western diet causes characteristic changes in the gut microbiome in the MAFLD and MAFLD-HCC murine models. **(A)** Linear discriminant analysis (LDA) effect size (LEfSe) was used to compare fecal samples between normal (ND)- and Western diet (WD)-fed wild type (WT) mice at the phylum and genus levels in the **(A, C)** MAFLD model (treated with acetone (AC)) and **(B, D)** MAFLD-HCC model (treated with DMBA). *Actinobacteriota* and *Desulfobacterota* are the common taxa upregulated by the WD in both animal models. At the genus level, more than 15 common changes between the models were detected.

In the next step, we compared WT animals subjected to the MAFLD and MAFLD-HCC models that were fed either a ND or WD. We observed a significant increase in the levels of *Actinobacteriota*, *Deferribacterota* and *Desulfobacterota*, while there was a decrease in *Cyanobacteria*, *Patescibacteria* and *Campilobacterota* in the animal model subjected to the MAFLD model after being fed WD (**Figure 4A**). Animals in the MAFLD-HCC model showed a higher abundance of phyla *Firmicutes*, *Actinobacteriota*, and *Desulfobacterota*. Conversely, WD-fed animals had lower abundances of *Cyanobacteria*, *Patescibacteria* and *Bacteroidota* compared to ND-fed animals (**Figure 4B**).

A comparison at the genus level revealed 44 differentially abundant genera in the MAFLD model. Out of these, 16 were enriched in the WT-WD group, while 28 were more abundant in the WT-ND group (**Figure 4C**). In the MAFLD-HCC model, 15 bacterial genera were enriched, and 26 genera had decreased abundances in the WT-WD group compared to the WT-ND group (**Figure 4D**). A total of 27 genera were detected in WT animals that showed alterations in abundance in both the MAFLD and MAFLD-HCC models. The genera enriched in the WT-WD group were *Bacteroides, Faecalibaculum, Lachnospiraceae GCA-900066575, Lactococcus, Romboutsia, Tuzzerella, unknown Atopobiaceae*, and *unknown Desulfovibrionaceae*. The genera that were reduced in abundance were *Odoribacter, Muribaculum, unknown Muribaculaceae, Alloprevotella, Prevotellaceae UCG-001, Alistipes, unknown Gastranaerophilales, Anaeroplasma, unknown RF39, unknown Clostridia UCG-014, unknown Clostridia vadinBB60 group, Eubacterium xylanophilum group, Lachnospiraceae NK4A136 group, Lachnospiraceae UCG-001, Roseburia, Butyricicoccus, Butyricicoccaceae UCG-009, Incertae Sedis*, and *Candidatus saccharimonas* (**Supplementary Table S3**). The taxa whose increased abundance was observed only in the MAFLD model were *Blautia*, *Rikenellaceae RC9 gut group*, *Parabacteroides*, *Lachnospiraceae UCG-006*, *Clostridium sensu stricto 1*, *Halomonas*, and *Alcaligenes*. Changes observed only in the MAFLD-HCC model were increased abundances of *Dubosiella*, *Lachnoclostridium*, *unknown Peptococcaceae*, *Desulfovibrio*, *Oscillospiraceae NK4A214 group*, and *Escherichia-shigella*.

### 3.3 *Plin5* deficiency reshapes the gut microbiome in the MAFLD model

**Figure 5:**
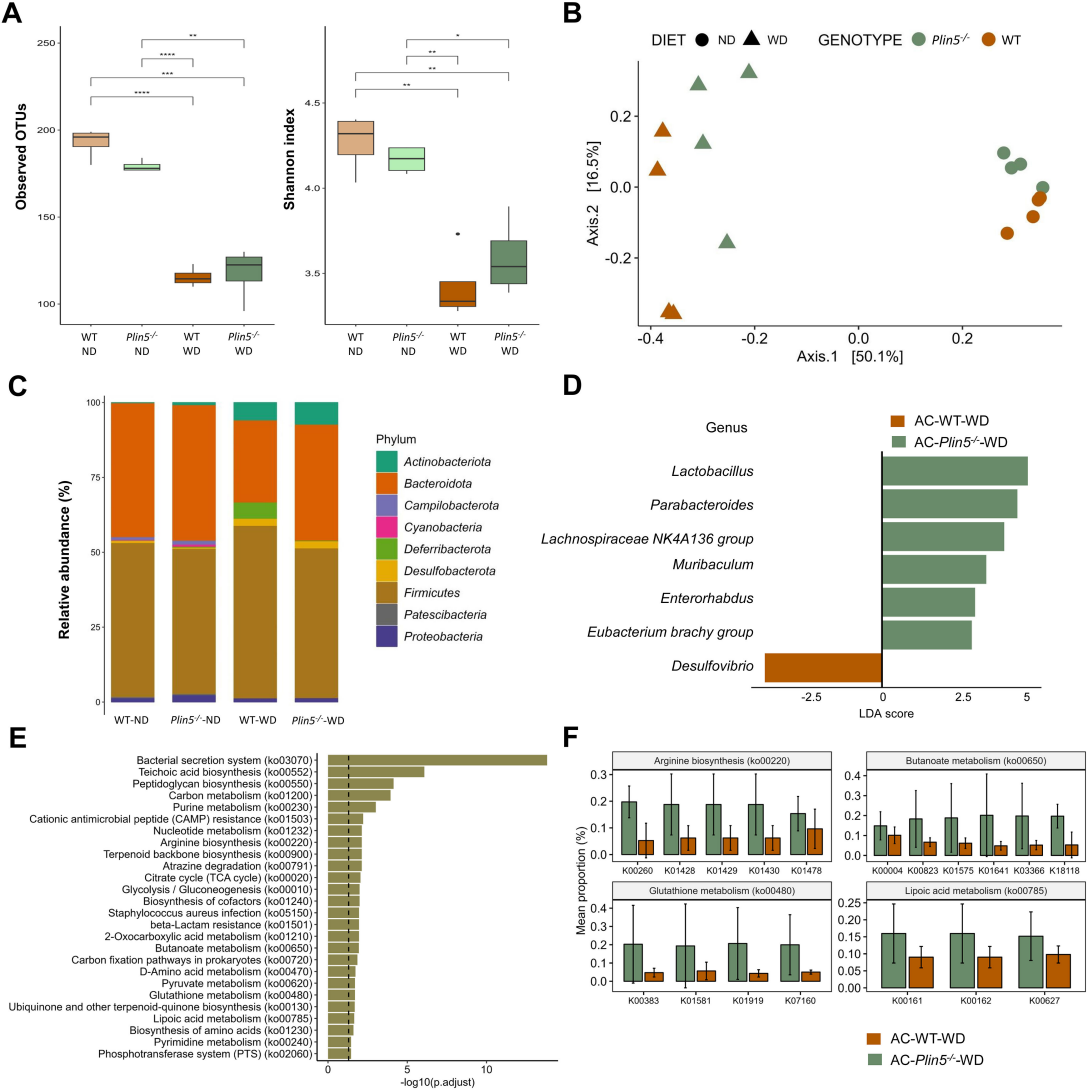
Fecal microbiome composition differs between WT and *Plin5^-/-^* mice in the MAFLD model. **(A)** Assessment of fecal microbiota α-diversity using observed operational taxonomic units (OTUs) (*left*) and Shannon diversity index (*right*) detected a statistically significant reduction in microbial diversity between the normal (ND)- and Western (WD)-fed groups. **(B)** Principal-coordinate analysis (PCoA) of a Bray-Curtis distance generated from fecal bacterial taxa. The four groups are marked as follows: Wild type (WT)-ND: brown points; *Plin5^-/–^* ND: green points; WT-WD: brown triangles; *Plin5^-/–^* WD: green triangles. **(C)** Average relative abundance analysis of fecal samples from the four groups at the phylum level. **(D)** Linear discriminant analysis effect size (LEfSe) and linear discriminant analysis (LDA) based on operational taxonomic units were used to differentiate between WT-WD and *Plin5^-/–^* WD taxa at the genus level. **(E)** Over-representation analysis (ORA) of significantly over-represented PICRUSt2 KEGG pathways (FDR < 0.05). The vertical line represents *p*-value cut-off of 0.05. **(F)** Mean proportion of KEGG Orthology (KO) terms with error bars of selected pathways involved in prevention of hepatocarcinogenesis from ORA. The significant KO were detected across of significant KEGG terms defined at *p*-value of 0.1 (Wilcoxon rank sum). Error bars represent the standard deviation (SD) calculated across all biological replicates. Abbreviation are: acetone (AC), normal diet (ND), wild type (WT), linear discriminant analysis (LDA). *: *p* < 0.05, **: *p* ≤ 0.01, ***: *p* ≤ 0.001, ****: *p* ≤ 0.0001.

To determine the impact of *Plin5* deficiency on the microbiome during MAFLD and MAFLD-HCC pathologies, we compared *Plin5^-/-^* animals treated with either AC or DMBA and fed a WD to corresponding WT controls. Firstly, we analyzed microbial α-diversity in the MAFLD model and found significant differences between ND- and WD-fed mice (**Figure 5A**). In the MAFLD model, we detected significantly higher observed OTUs and Shannon indices for species in both WT and *Plin5^-/-^* mice on ND compared to mice fed WD, indicating a reduced microbiome diversity caused by WD. Importantly, we did not observe any significant differences in observed OTUs and Shannon indices between genotypes with the same dietary conditions (WT-ND *vs*. *Plin5^-/^*-ND; WT-WD *vs. Plin5^-/–^* WD). Nevertheless, we found a non-statistically significant tendency towards higher diversity after WD in *Plin5^-/-^* mice compared to WT mice.

Subsequently, β-diversity was assessed and presented as a principal coordinate analysis (PCoA) ordination based on the Bray-Curtis dissimilarity matrix. The PCoA graph clearly showed a separation of all four animal groups in the MAFLD model (WT-ND, WT-WD, *Plin5^-/–^* ND, and *Plin5^-/–^* WD). The most significant clustering was observed on the x-axis, where a clear distinction between clusters representing animals fed ND (on the right) and animals fed WD (on the left) accounted for 50.1% of the differences in microbial composition of the feces (**Figure 5B**). Two distinct clusters, WT-WD and *Plin5^-/–^* WD, were still present after WD, suggesting a possible role of *Plin5* deficiency in the regulation of the microbiome during MAFLD pathology. Additionally, separate clustering of WT-ND and *Plin5^-/–^* ND was observed on the y-axis, confirming that the loss of *Plin5* is responsible for 16.1% of the microbial diversity.

Moreover, the analysis of fecal microbiota abundance in ND-fed animals revealed that the most common bacterial phyla in WT and *Plin5^-/-^* were *Actinobacteriota*, *Bacteroidota*, *Firmicutes*, *Proteobacteria* and *Campilobacterota*, respectively (**Figure 5C**). When switched to a WD, *Plin5^-/-^* animals, like WT animals, showed a decrease in the relative abundances of *Campilobacterota*, *Cyanobacteria* and *Patescibacteria* as well as an increase in *Actinobacteriota* and *Desulfobacterota* levels (**Supplementary Figure S1A**). However, the increase in *Deferribacterota* (cf. **Figure 4B**), which was observed in WT-WD animals, was not detected in the *Plin5^-/–^* WD group. Therefore, similar changes at the phylum level were observed in both WT and *Plin5^-/-^* animals in the MAFLD model.

We performed LEfSe on multiple taxonomical levels and discovered differences in 7 bacterial taxa at the genus level (**Figure 5D**). The group of WT-WD animals exhibited a significantly higher abundance of *Desulfovibrio* compared to *Plin5^-/–^* WD. Conversely, the loss of *Plin5* resulted in an upregulation of *Lactobacillus, Parabacteroides, Lachnospiraceae NK4A136 group, Muribaculum, Enterorhabdus*, and *Eubacterium brachy group*.

Furthermore, KEGG pathway analysis revealed the deregulation of several biological pathways following *Plin5* ablation. The top five pathways, in terms of statistical significance, were clustered around the bacterial secretion system, teichoic acid biosynthesis, peptidoglycan biosynthesis, carbon and purine metabolism (**Figure 5E**). From the over-represented KEGG pathways we selected those with known role in stopping or slowing progression from steatosis to hepatocarcinogenesis (**Figure 5F**) and all of the genes are more increased in *Plin5^-/–^* WD animals than in WT-WD.

### 3.4 *Plin5* deficiency alters the microbial composition in a MALFD-HCC model

**Figure 6:**
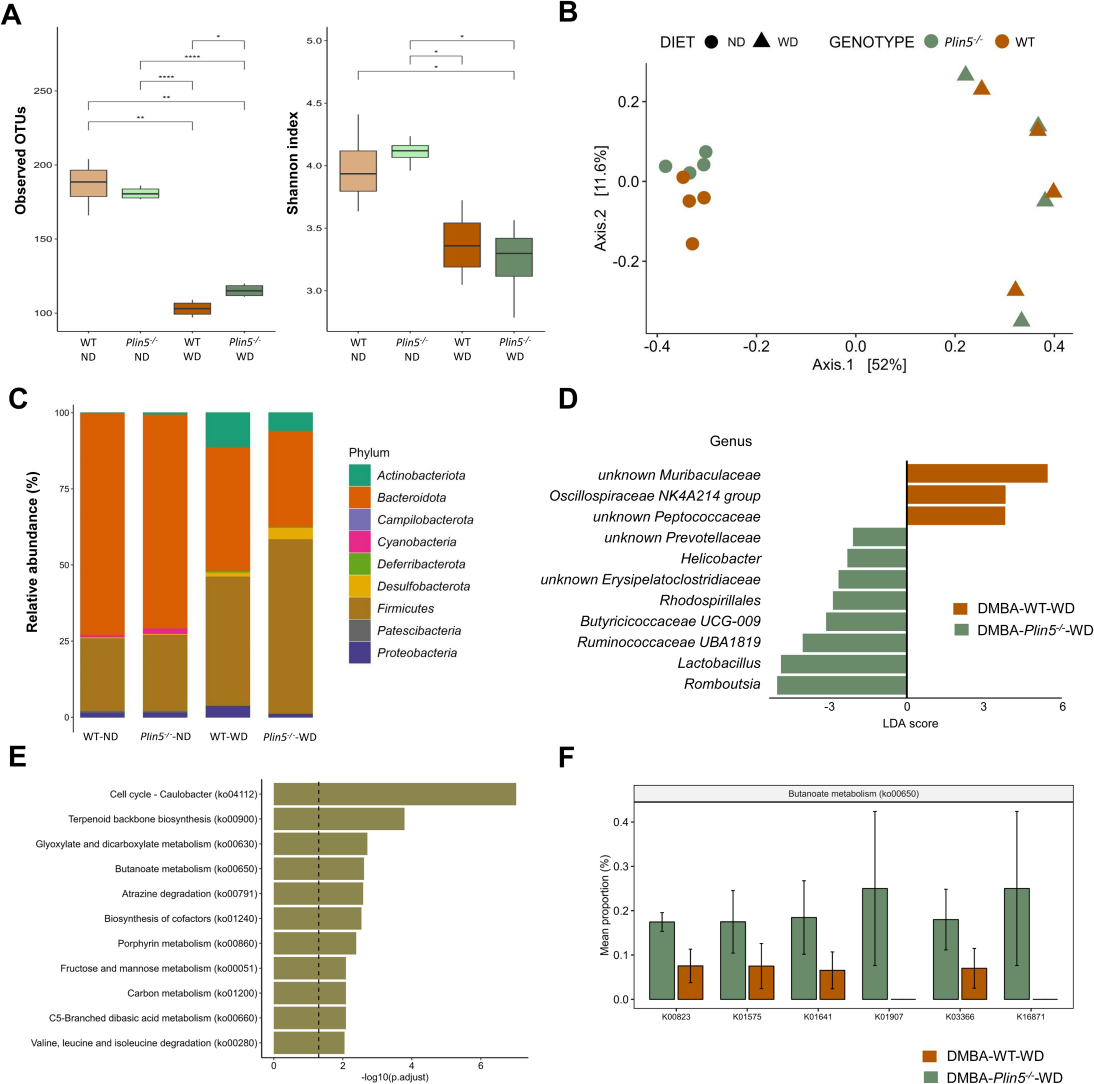
Fecal microbiome composition differs between WT and *Plin5^-/-^* mice in the MAFLD-HCC model. **(A)** α-diversity assessment of fecal microbiota using Observed OTUs (*left*) and Shannon diversity (*right*) index detected a statistically significant reduction in microbial diversity between ND and WD-fed groups of MAFLD-HCC. **(B)** Principal-coordinate analysis (PCoA) of a Bray-Curtis distance generated from fecal bacterial taxa. Four groups are marked as follows: WT-ND: brown points; *Plin5^-/–^* ND: green points; WT-WD: brown triangles; *Plin5^-/–^* WD: green triangles. **(C)** Average relative abundance analysis of fecal samples from the four groups at the phylum level. **(D)** Linear discriminant analysis effect size (LEfSe) and linear discriminant analysis (LDA) based on operational taxonomic units were used to differentiate between WT-WD and *Plin5^-/–^* WD taxa at the genus level. **(E)** Over-representation analysis (ORA) of significantly over-represented PICRUSt2 KEGG pathways (FDR < 0.05). The vertical line represents *p*-value cut-off of 0.05. **(F)** Mean proportion of KEGG Orthology (KO) terms with error bars of selected pathways involved in prevention of hepatocarcinogenesis from ORA. The significant KO were detected across significant KEGG terms defined at a *p*-value of 0.1(Wilcoxon rank sum). Error bars represent the standard deviation (SD) calculated across all biological replicates. Abbreviations used are: operational taxonomic unit (OTU), 7,12-Dimethylbenz(a)anthracene (DMBA), normal diet (ND), wild type (WT), linear discriminant analysis (LDA). *: *p* < 0.05, **: *p* ≤ 0.01, ****: *p* ≤ 0.0001.

The same analysis was performed on the sequencing data from the MAFLD-HCC model, as was done for the MAFLD model. Firstly, the fecal microbiota diversity of WT and *Plin5^-/-^* animals in the MAFLD-HCC model was assessed through α-diversity analysis using observed OTUs and Shannon. The data showed that the diversity of the fecal microbiome tended to decrease after WD in both WT and *Plin5^-/-^* animals, as indicated by the median values of observed OTUs and Shannon diversity index (**Figure 6A**). The reduction in observed OTUs was statistically significant for both WT and *Plin5^-/-^* animals, while Shannon showed only a tendency towards reduced diversity. Importantly, the microbial diversity was significantly higher in *Plin5^-/-^* WD-fed animals compared to WT WD-fed animals.

PCoA ordination, based on a Bray-Curtis dissimilarity matrix, displayed a distinct separation of various animal groups (**Figure 6B**). On the x-axis, there was a differentiation of clusters representing animals fed a ND on the left, compared to those fed a WD on the right. This difference accounted for 52% variation in microbial diversity, indicating that the most significant variations in microbial composition between the four groups were caused by the WD. Additionally, there was a noticeable separation of animals based on their genotype on the y-axis, which explained an 11.6% difference in diversity between the WT-ND and the *Plin5^-/–^* ND group. However, this clear separation in gut microbial composition between WT and *Plin5* ^-/-^ genotypes disappeared after the introduction of the WD. Remarkably the difference in fecal microbial composition was more pronounced between animal groups with different diets than between groups with different genotypes.

When assessing the abundance of bacterial taxa at the phylum level, we found that *Bacteroidota*, *Firmicutes* and *Proteobacteria* were the dominant phyla in WT-ND and *Plin5^-/–^* ND animals (**Figure 6C**). The relative abundance of *Campilobacterota*, *Deferribacterota*, *Desulfobacterota*, *Actinobacteriota*, and *Firmicutes* increased in *Plin5^-/-^* animals after WD (**Supplementary Figure S1**). Compared to WT-WD animals, the *Plin5^-/–^* WD group showed a statistically significant increase in the abundance of *Campilobacterota* (**Supplementary Figure S1**).

To further explore the difference in microbial composition between groups, LefSe analysis at the genus level was performed, revealing several statistically significant differences among the four groups (**Figure 6D**). Notably, the *Plin5^-/–^* WD group had significantly higher abundances of *Lactobacillus* and *Romboutsia*, along with a slightly lower increase in *Prevotellaceae*, *Helicobacter*, *Erysipelatoclostridiaceae*, *Rhodospirillales*, *Butyricicoccaceae UCG-009*, and *Ruminococcaceae UBA1819*.

Lastly, KEGG analysis showed deregulation of several terms in *Plin5* null mice, with the top five in significance being cell cycle–Caulobacter, terpenoid backbone synthesis, glyoxylate and dicarboxylate metabolism, butanoate metabolism and atrazine degradation (**Figure 6E**). *Plin5^-/–^* WD animals have significantly increased butanoate metabolism (**Figure 6F**) compared to WT-WD in MAFLD-HCC model.

### 3.5 The microbiome of MAFLD and MAFLD-HCC models in WT mice differs in bacterial taxa at the genus level

**Figure 7:**
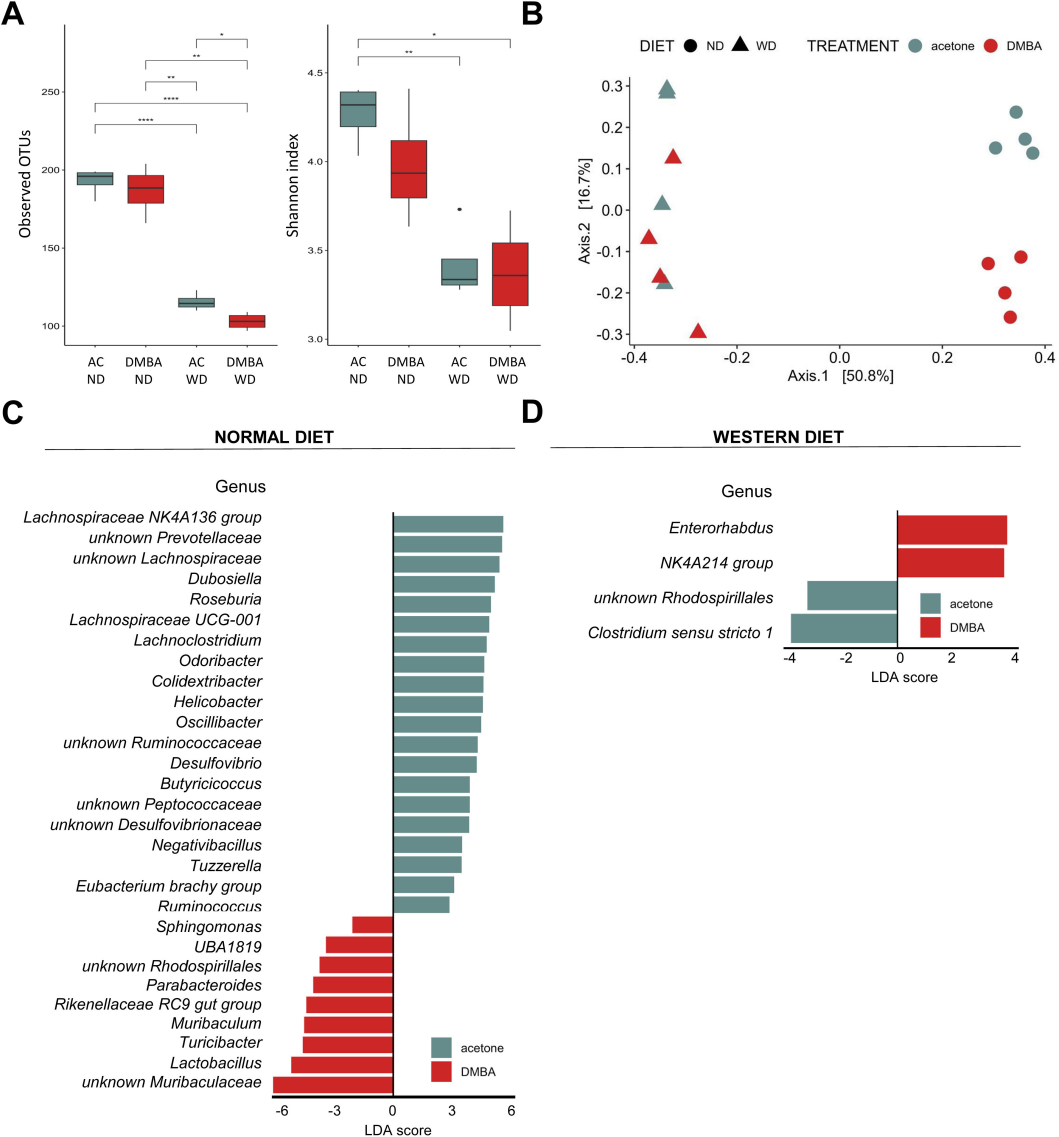
Fecal microbiome composition differs between the MAFLD and MAFLD-HCC models in WT mice. **(A)** α-diversity assessment of fecal microbiota using Observed OTUs (*left*) and Shannon diversity (*right*) index between the four animal groups. Groups are: AC-ND, animals that received acetone and a normal diet; DMBA*-*ND, animals that received DMBA and a normal diet; AC-WD, animals that received acetone and a Western diet; DMBA*-*WD, animals that received DMBA and a Western diet. **(B)** Principal-coordinate analysis (PCoA) of a Bray-Curtis distance generated from fecal bacterial taxa. The four groups are marked as follows: acetone-ND: cyan points; DMBA*-*ND: red points; acetone-WD: cyan triangles; DMBA*-*WD: red triangles **(C, D)** Differences in microbiome at the phylum and genus levels between the different groups subjected either to **(C)** normal or **(D)** Western diet. *: *p* < 0.05, **: *p* ≤ 0.01, ****: *p* ≤ 0.0001.

Finally, we conducted a search for differences in bacterial composition between fecal samples from WT animals belonging to the MAFLD and MAFLD-HCC model. The PCoA analysis showed clustering of three groups: DMBA-ND, AC-ND, and a cluster consisting of WD-fed animals from both animal models (**Figure 7A**). We see reduction in both Observed OTUs and Shannon index in groups fed a WD was observed (**Figure 7B**). Interestingly, while no statistically significant difference in Shannon index was observed between WD-fed groups, DMBA-WD group had significantly lower level of Observed OTUs. Additionally, the LDA analysis on the phylum level revealed that *Firmicutes*, *Campilobacterota* and *Desulfobacterota* were enriched in the AC-ND group compared to the DMBA-ND group, while *Bacteroidota* were enriched in the DMBA-ND group (**Supplementary Figure S2**). Interestingly, there were no differences in microbiome at the phylum level between the AC- and DMBA-treated WT-WD groups. However, more differences were detected at the genus level, with the ND-fed groups differing in 29 bacterial taxa and the WD-fed animals differing in four taxa (**Figures 7C, D**). Specifically, WD-fed animals in the MAFLD-HCC model had a high abundance of *Enterorhabdus* and *Oscillospiraceae NK4A214 group*, while the abundance of *unknown Rhodospirillales* and *Clostridium sensu stricto 1* was enriched in animals of the MAFLD model.

## 4 Discussion

Microbiota plays a fundamental role in gut and liver health (Schwenger et al., 2019). Significant changes in the microbial composition of the gut have been associated with the development and progression of liver diseases (Hrncir et al., 2021), and specifically in the pathogenesis of HCC and the shaping of the tumor microenvironment (Chen et al., 2023; Liu et al., 2023). In this study, we aimed to characterize the microbial changes in the gut during the pathology of MAFLD and MAFLD-HCC in two murine models. Additionally, we assessed the impact of *Plin5* deficiency on the gut microbiome in the same models.

Firstly, it was assumed that the loss of *Plin5* would only alter microbial composition in WD conditions, based on the fact that *Plin5^-/-^* animals do not differ from WT mice when fed with ND (Wang et al., 2015; Kuramoto et al., 2012). However, this assumption was disproven in both MAFLD and MAFLD-HCC models through LDA scoring, which detected differences in several bacterial taxa between ND-fed WT and knockout animals. Additionally, PCoA analysis showed that ND-fed WT and *Plin5^-/-^* animals clustered separately. Interestingly, *Plin5^-/-^* animals in both models had an increased abundance of *Actinobacteriota* that play a pivotal role in maintaining intestinal homeostasis (Binda et al., 2018). At the genus level, *Atopobiaceae*, that promote cardiometabolic health (Vinke et al., 2017), displayed increased abundance in *Plin5^-/-^* animals in both models. Furthermore, *Alloprevotella*, which has been found to be significantly enriched in fecal samples of patients with irritable bowel syndrome (Tang et al., 2023) was reduced in *Plin5^-/-^* mice. Taken together, the data suggests that *Plin5* is a critical factor that impacts the microbial gut content in healthy organisms.

Secondly, we analyzed the microbial changes observed in the MAFLD and MAFLD-HCC models. To do this, we compared WD-fed WT animals from both models to their corresponding ND-fed controls. As others have previously reported, the expected changes included an increase in the abundance of *Firmicutes* and *Proteobacteria*, along with a decrease in *Bacteroidota* (Murphy et al., 2015; Gupta et al., 2022). However, the MAFLD model did not exhibit the same changes. Interestingly, both models did show an increase in *Actinobacteriota* and *Desulfobacterota*, as well as a decrease in *Cyanobacteria* and *Patescibacteria*. Therefore, these common changes in *Actinobacteriota*, *Desulfobacterota*, *Cyanobacteria* and *Patescibacteria*, could be considered a general microbiome signature associated with fatty liver pathologies.

Furthermore, we confirmed that the diversity in microbial composition of feces was reduced after WD in both murine models, which was consistent with previous studies on MAFLD/MASH (Astbury et al., 2020; Jiang et al., 2023; Behary et al., 2021; Porras et al., 2017). Interestingly, unlike WT animals whose microbial diversity was significantly lower following WD, *Plin5*-deficient animals fed WD showed a tendency towards higher OTU diversity than WD-fed WT littermates. In the MAFLD-HCC model, the loss of *Plin5* in WD-fed mice preserved the level of OTU diversity compared to WD-fed WT animals, indicating that *Plin5* acts as a protective factor in preserving microbial diversity during fatty liver disease. In this regard, differences were observed between WD-fed WT and *Plin5-*deficient animals on several taxonomic levels in both models. While the most significant differences between the groups resulted from WD feeding in both models, WT and *Plin5-*null mice fed a ND showed more than 10% differences indicating that *Plin5* is indeed an important regulator of microbial gut composition.

**Figure 8:**
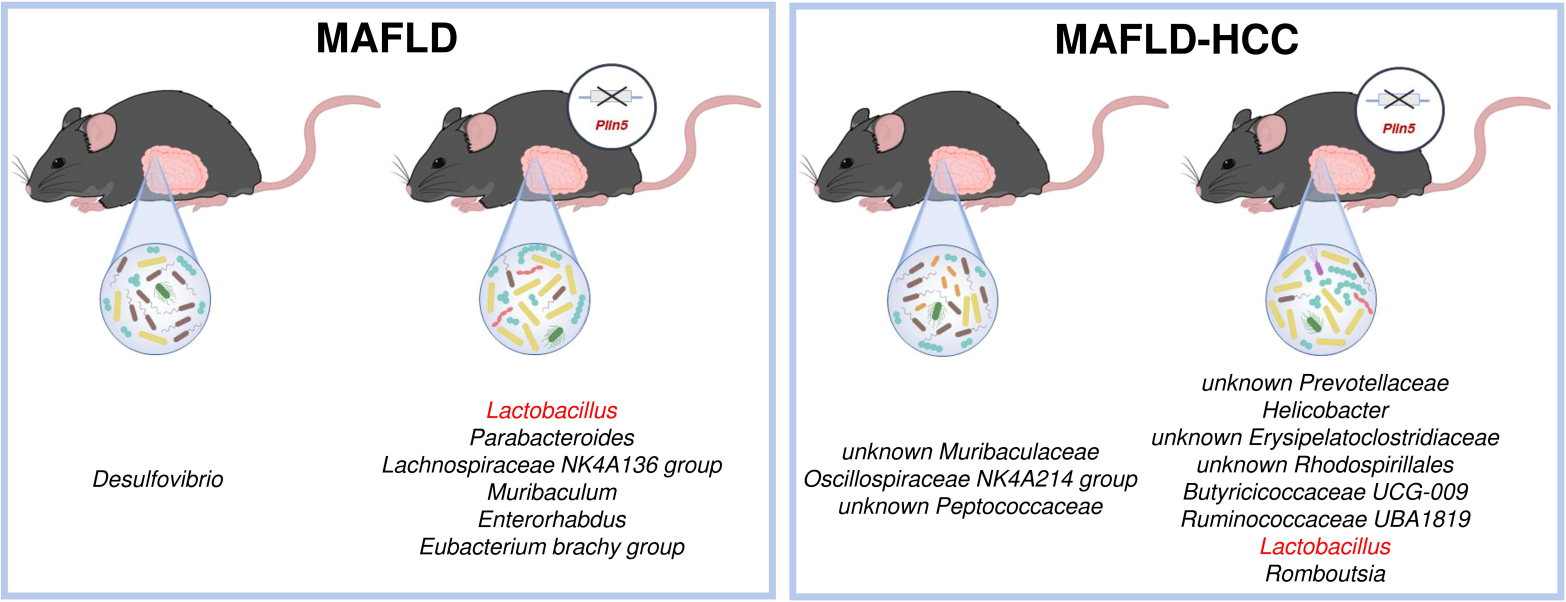
Schematic overview of the most significant differences between wild type and *Plin5* ^-/-^ mice in the MAFLD (*left*) or MAFLD-HCC (*right*) models. In both models, *Lactobacillus* is found in higher abundance in the *Plin5* ^-/-^ group compared to the wild type group.

The most significant difference found as a common denominator in both animal models is the significantly higher abundance of the *Lactobacillus* genus present in *Plin5-*deficient animals (**Figure 8**). Levels of *Lactobacillus* have been found to be increased in cirrhotic patients and MAFLD in multiple studies (Ponziani et al., 2019; Zhu et al., 2023). However, several reports have suggested that *Lactobacillus* acts as a protector of the intestinal barrier and a factor that attenuates the progression of MAFLD by lowering cholesterol and steatosis (Huang and Zheng, 2010; Yu et al., 2015; Lee et al., 2021; Li et al., 2021). In line with this, supplementation of *Lactobacillus lactis* and *Pediococcus pentosaceus* effectively normalized weight ratio, MAFLD activity score, biochemical markers, cytokine expression and gut-tight junction by modulating and reprogramming the gut metagenomic and metabolic environment, thus highlighting its protective effects (Yu et al., 2021). However, the exact mechanism of *Lactobacillus* regulation via PLIN5 still remains to be elucidated.

Furthermore, one study reported that *Lactobacillus* and *Bifidobacterium* improved hepatic steatosis and fibrosis in high-fat diet-fed mice. This improvement was linked to a decrease in the abundance of *Desulfovibri*o in feces, which aligns with the results we obtained in the MAFLD model (Lin et al., 2022). However, besides *Lactobacillus*, several other bacterial genera are enriched in *Plin5*-deficient animals in both models. Interestingly, increased abundances of *Romboutsia* have been detected *Plin5-*deficient animals in MAFLD-HCC model, despite their previous association with the severity of MAFLD and type 2 diabetes (Gu et al., 2022; Si et al., 2021). Similarly, there are conflicting results regarding the association between increased abundance of *Helicobacter* and MAFLD (Wijarnpreecha et al., 2018; Liu et al., 2021; Porras et al., 2017). In MAFLD model, *Parabacteroides* which show higher prevalence in cirrhotic patients, as well as an increase in MAFLD and MASH (Ponziani et al., 2019; Aron-Wisnewsky et al., 2020), are found in *Plin5*-deficient animals. In the case of *Enterorhabdus*, which is also abundant in *Plin5^-/–^* WD-fed animals, previous research has proven that it is enriched in healthy controls, rather than HCC patients (Bian et al., 2022). Consistent with our hypothesis that *Plin5* deficient animals on WD are healthier than WT controls, genera *Oscillospiraceae NK4A214* that has been identified previously as a prognostic marker for obesity, various metabolic disorders and inflammatory bowel diseases was more enriched in WT animals of the MAFLD model (Burakova et al., 2022).

For functional analysis, we identified changed metabolic pathways involved in lipid and carbohydrate metabolism such as terpenoid backbone synthesis, glyoxylate and dicarboxylate pathway and carbon metabolism in the MAFLD and MAFLD-HCC WD models when *Plin5* was knocked-out. Pathways associated with regulation of the tumor progression and microenvironment by exhibiting anti-inflammatory and anti-tumorigenic properties were more detected in *Plin5-*deficient animals. In both disease models we detected enrichment in butanoate metabolism in *Plin5-*deficient compared to WT animals. Butanoate or butyrate metabolism is one of important metabolism involved in production of short chain fatty acids (SCFAs), butyrate. Compelling evidence through multiple studies have shown that increase in butyrate-producing bacteria, such as *Lachnospiraceae NK4A136 group*, which we found in this study in the MAFLD model, and butyrate production attenuates steatohepatitis by improving intestinal barrier function, up-regulating glucagon-like peptide-1 receptor (GLP-1R) expression, and down-regulating inflammatory signaling as well as oxidative damage in the liver (Zhu et al., 2023; Zhou et al., 2017, 2018; Jin et al., 2015; Endo et al., 2013). Overall, we detect more antioxidant enriched pathways in *Plin5-*deficient animals in MAFLD than MAFLD-HCC animal models. For instance, antioxidant enriched pathway in *Plin5-*deficient animals was lipoic acid metabolism. It has been shown that lipoate acts as antioxidants either by radical quenching or indirectly by recycling other antioxidants such as vitamin C, vitamin E, coenzyme Q10, and glutathione (Spalding and Prigge, 2010; Kagan et al., 1992; Scholich et al., 1989; Xia et al., 2001; Jocelyn, 1967). We also found glutathione metabolism to be increased in *Plin5-*deficient animals. Another interesting pathway increased in *Plin5-*deficient animals within the MAFLD model was arginine biosynthesis. Recent findings highlighted that increased production of l-arginine by *Lactobacillus plantarum* was a good potential treatment for NAFLD (Kim et al., 2023). Taken together these results imply a positive shift in microbial metabolic pathways towards reducing progression and attenuating more predominantly the MAFLD state but also the MALFD-HCC states.

However, as we are lacking detailed serum measures of circulating bacterial products and mechanistic insight regarding the mode of action of each bacterial genera in our models, it is challenging to conclude whether increase in particular bacterial taxa is a body’s defense mechanism or opportunistic bacteria spreading with detrimental effects especially for certain taxa with conflicting literature results. Therefore, it would be of utmost importance to focus future research on characterizing the impact of particular bacterial taxa on the progression of MAFLD or HCC.

Lastly, we did not observe any significant variations in bacterial composition in WT animals fed a WD and subjected to the two models. Although the models differ in four bacterial genera, namely *Enterorhabdus*, *Oscillospiraceae NK4A214 group, unknown Rhodospirillales*, and *Clostridium sensu stricto* 1, none of them were exclusively associated with MAFLD or MAFLD-HCC. In summary, the data suggests that the MAFLD and MAFLD-HCC models exhibit similar microbial signatures, making it difficult to distinguish between them.

While this study did not provide a mechanistic insight into how the absence of *Plin5* affects the gut microbiome, another member of the perilipin family has been examined in the context of diet-induced changes in the microbiome. The study indicated that the removal of perilipin 2 (*Plin2*) alters the expression of microbial enzymes, leading to the production of energy and components necessary for cell growth (Xiong et al., 2017). Given the similarities in function and structure between *Plin2* and *Plin5*, we hypothesized that a similar mechanism could explain the observed changes in the microbiome of *Plin5* ^-/-^ mice. However, limitations of this study, such as the absence of metatranscriptomics, could be utilized to further understand the genotype-dependent changes observed.

Dietary intervention in murine models has been shown to affect microbiome composition, demonstrating similarities with human studies (Nguyen et al., 2015). Therefore, our report can serve as a reference for a better understanding of microbiome composition in liver pathologies associated with MAFLD. Additionally, we have conducted a thorough analysis that indicates minimal differences between our models, emphasizing the importance of distinguishing between HCC cases of MAFLD and non-MAFLD origin. Despite the challenges, future research should prioritize human studies to investigate host-microbiota interactions and elucidate the role of *Plin5*. This research could be instrumental in the development of future therapeutic approaches in liver pathologies.

Our current data may not directly translate to clinical implications, but it does provide insight into potential therapeutic options for liver disease. One discovery we made is that *Plin5* plays a role in disrupting the microbiome population in both MAFLD and HCC. This suggests that developing pharmacological inhibitors or knockout therapies targeting Plin5 could help prevent the advancement of liver disease. Additionally, our analysis of the microbiome in two liver pathologies revealed that the *Lactobacillus* genus may have a beneficial impact on the progression of MAFLD to HCC. A recent study also found that supplementing *Lactobacillus acidophilus* can suppress NAFLD to HCC progression in mouse models (Lau et al., 2024). However, further research involving human samples and cohorts is needed to confirm these findings.

## 5 Conclusions

This study identifies PLIN5 as a crucial regulator of gut microbiota composition during the development of MAFLD and its progression to HCC. Our findings demonstrate that *Plin5* deficiency leads to significant shifts in gut microbiota, including an increase in beneficial taxa like *Lactobacillus*, which has been linked to improved liver function and reduced disease severity. Moreover, the Western diet exacerbated microbial alterations, indicating the complex interaction between diet, genetic factors, and the microbiome in liver disease progression. The broader implications of these findings suggest that targeting PLIN5 could serve as a potential therapeutic strategy to modulate gut microbiota and mitigate liver disease progression. By identifying specific microbial signatures linked to *Plin5* deficiency, this study provides a foundation for future research exploring gut-liver interactions and their impact on MAFLD and MAFLD-HCC. These insights are valuable not only for understanding the pathophysiology of liver diseases but also for developing targeted microbiome-based therapies. However, despite these insights, the study has notable limitations that should be considered when interpreting the results. A key limitation is the absence of bacterial metabolites analysis, which restricts our ability to fully understand the functional implications of the observed microbial changes. Without direct measurement of metabolites, such as short-chain fatty acids or bile acids, the exact mechanisms by which *Plin5* influences liver disease through gut microbiota remain speculative.

In conclusion, the study underscores the importance of *Plin5* as a key regulator of gut microbiota in the context of liver disease and highlights the potential of microbiome modulation as a therapeutic avenue. Future research should focus on validating these findings in human studies and exploring the therapeutic potential of manipulating gut microbiota to prevent or treat liver disease.

## Data Availability

The raw microbiome 16S rRNA sequencing data generated in this study have been deposited in the Sequence Read Archive (SRA) under the accession number PRJNA1157013.

## Supplementary material

Supplementary Figure 1 Abundances of bacterial taxa.

Supplementary Figure 2 Linear discriminant analysis (LDA) at the phylum level.

Supplementary Table 1 Changes in number of detected OTUs before and after each IMNGS pipeline filtering step.

Supplementary Table 2 Changes in shared bacterial taxa caused by *Plin5^-/-^* deletion in normal diet-fed mice of MAFLD and MAFLD-HCC models.

Supplementary Table 3 Changes in shared bacterial taxa in wild type animals subjected to the MAFLD and MAFLD-HCC models.
