# Supplementary material for: The influence of perilipin 5 deficiency on gut microbiome profiles in murine metabolic dysfunction-associated fatty liver disease (MAFLD) and MAFLD-hepatocellular carcinoma
Source: Front Cell Infect Microbiol. 2024 Oct 14;14:1443654. doi: 10.3389/fcimb.2024.1443654 (PMC11513398; doi:10.3389/fcimb.2024.1443654)
Supplement: Supplementary Figure 1 — Abundances of bacterial taxa. [file DataSheet1.pdf]

Supplementary Material

1 Supplementary Figures and Tables

1.1 Supplementary Figures

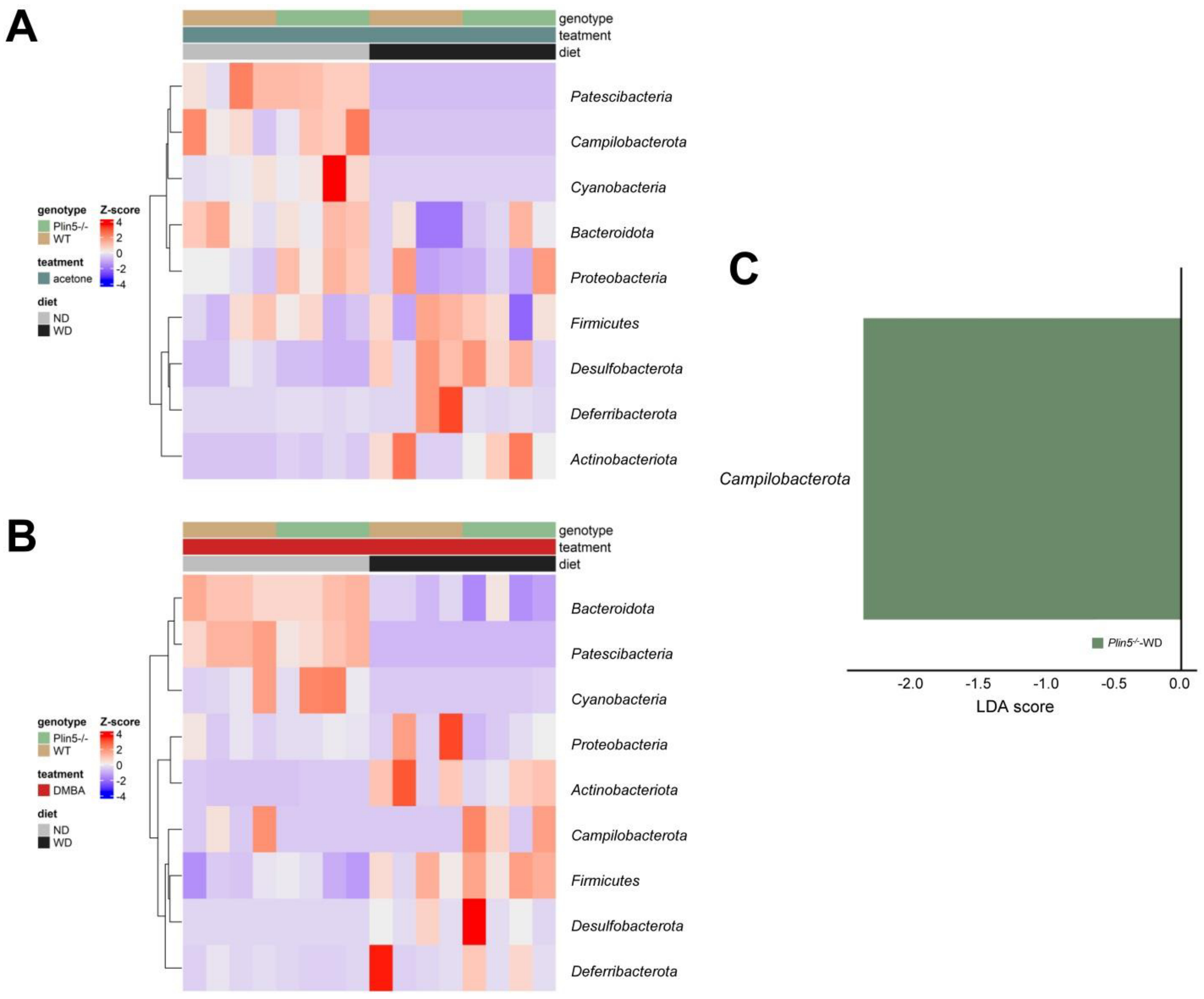

Suppl. Figure 1. Abundances of bacterial taxa. This figure displays heatmaps that depict the abundances of bacterial taxa at the phylum level for **(A)** the MAFLD and **(B)** the MAFLD-HCC models. **(C)** Results of the LEfSe analysis comparing WT-WD to *Plin5*$^{-/-}$-WD animals of the MAFLD-HCC model. Abbreviations: normal diet (ND); Western diet (WD); wild type (WT); linear discriminant analysis (LDA).

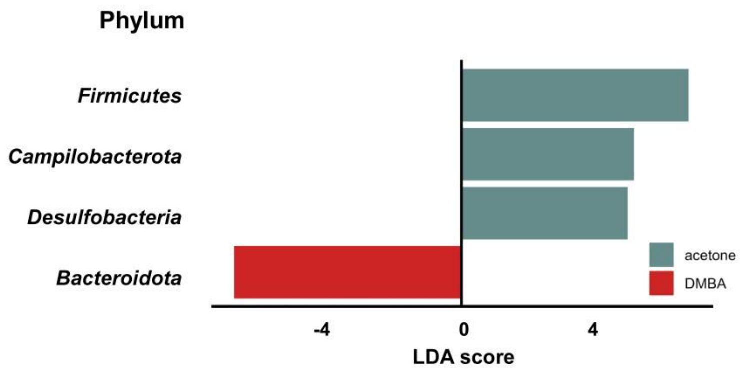

Suppl. Figure 2. Linear discriminant analysis (LDA) at the phylum level. The LDA effect size (LEfSe) was used to compare taxa at the phylum level in fecal samples between normal diet (ND) fed wild type (WT) animals treated with either acetone or DMBA. *Firmicutes*, *Campilobacterota*, *Desulfobacteria* were more abundant in acetone treated animals, while *Bacteroidota* were more abundant in DMBA treated mice.

1.2 Supplementary Tables

Suppl. Table 1. Changes in number of detected OTUs before and after each IMNGS pipeline filtering step.

sample_id	Animal number	GROUP	Demultiplexing	Merging	EE-filtering	Chimeras -Artifacts	OTU Abundance filter
Sample01	954_4	WT ND acetone	65927	64754	54708	51491	49889
Sample02	947_5	WT ND acetone	48435	47496	39749	37504	36749
Sample03	948_6	WT ND acetone	47800	46923	39462	37518	36312
Sample04	518_7	WT ND acetone	41137	40406	33940	32338	31335
Sample09	370_24	*Plin5*$^{-/-}$ ND acetone	25717	25176	21126	20076	19639
Sample10	371_25	*Plin5*$^{-/-}$ ND acetone	50566	49593	41728	39265	38266
Sample11	376_26	*Plin5*$^{-/-}$ ND acetone	59665	58451	49096	46682	45898
Sample12	377_27	*Plin5*$^{-/-}$ ND acetone	29357	28752	24034	23480	23014
Sample13	429_33	WT ND DMBA	50005	48916	40905	39396	39075
Sample14	430_34	WT ND DMBA	51946	50858	42842	40549	39800
Sample15	444_35	WT ND DMBA	43686	42851	36084	33951	33459
Sample16	445_36	WT ND DMBA	57090	55725	46383	42056	40977
Sample21	454_53	*Plin5*$^{-/-}$ ND DMBA	30732	30205	25273	24926	24502
Sample22	458_54	*Plin5*$^{-/-}$ ND DMBA	16158	15814	13150	13067	12814
Sample23	459_55	*Plin5*$^{-/-}$ ND DMBA	27360	26626	22171	21842	21500
Sample24	466_56	*Plin5*$^{-/-}$ ND DMBA	30924	30114	25173	24964	24623
Sample25	956_64	WT WD acetone	35414	34565	28508	28196	27847
Sample26	949_65	WT WD acetone	30159	29450	24302	24184	24082
Sample27	4_67	WT WD acetone	29220	28699	23573	23348	23104
Sample28	5_68	WT WD acetone	38885	38275	32064	31655	31424
Sample33	372_84	*Plin5*$^{-/-}$ WD acetone	33950	33427	28210	27768	27362
Sample34	373_85	*Plin5*$^{-/-}$ WD	42071	41244	34427	34270	33986

		acetone					
Sample35	378_86	*Plin5*$^{-/-}$ WD acetone	30698	29892	24490	24192	24019
Sample36	382_88	*Plin5*$^{-/-}$ WD acetone	17282	16643	11824	11752	11638
Sample37	432_94	WT WD DMBA	38396	37559	30941	30648	30393
Sample38	446_95	WT WD DMBA	24805	23983	19590	19519	19409
Sample39	453_96	WT WD DMBA	23492	22965	18836	18741	18511
Sample40	475_98	WT WD DMBA	33417	32652	27088	26928	26648
Sample45	455_13	*Plin5*$^{-/-}$ WD DMBA	31000	30553	25624	25420	25145
Sample46	461_15	*Plin5*$^{-/-}$ WD DMBA	33645	33066	27878	27827	27668
Sample47	468_16	*Plin5*$^{-/-}$ WD DMBA	34614	33958	28367	28193	27976
Sample48	483_18	*Plin5*$^{-/-}$ WD DMBA	38422	37323	30722	30410	30293
SampleNC	blank extraction	blank extraction	578	329	202	201	201

Abbreviations used: DMBA, 7,12-dimethylbenz(a)anthracene; ND, normal diet; *Plin5*$^{-/-}$, mice lacking Perilipin 5; WD, Western diet; WT, wild type.

Suppl. Table 2. Changes in shared bacterial taxa caused by *Plin5*$^{-/-}$ deletion in normal diet-fed mice of MAFLD and MAFLD-HCC models.

**Figure 3.**<br>***Plin5*$^{-/-}$ *vs*. wild type (WT) animals (normal diet)**	
**Phylum**	**Abundantly present in**
Actinobacteriota	*Plin5*$^{-/-}$ mice
**Genus**	**Abundantly present in**
unknown Atopobiaceae	*Plin5*$^{-/-}$ mice
Alloprevotella	WT mice

Suppl. Table 3. Changes in shared bacterial taxa in wild type animals subjected to the MAFLD and MAFLD-HCC models.

**Figure 4.**<br>**Normal diet (ND)- *vs*. Western diet (WD)-fed animals (wild type)**	
**Phylum**	**Abundantly present in**
Actinobacteriota	WD-fed mice
Desulfobacterota	WD-fed mice
Patescibacteria	ND-fed mice
Cyanobacteria	ND-fed mice
**Genus**	**Abundantly present in**
Alistipes	ND-fed mice
Alloprevotella	ND-fed mice
Anaeroplasma	ND-fed mice
Butyricicoccus	ND-fed mice
Candidatus saccharimonas	ND-fed mice
Eubacterium xylanophilum group	ND-fed mice
Incertae Sedis	ND-fed mice
Lachnospiraceae NK4A136 group	ND-fed mice
Lachnospiraceae UCG-001	ND-fed mice
Muribaculum	ND-fed mice
Odoribacter	ND-fed mice
Prevotellaceae UCG-001	ND-fed mice
Roseburia	ND-fed mice
UCG-009	ND-fed mice
unknown Clostridia UCG-014	ND-fed mice
unknown Clostridia vadinBB60 group	ND-fed mice
unknown Gastranaerophilales	ND-fed mice
unknown Muribaculaceae	ND-fed mice
unknown RF39	ND-fed mice
Bacteroides	WD-fed mice
Faecalibaculum	WD-fed mice
GCA-900066575	WD-fed mice
Lactococcus	WD-fed mice
Romboutsia	WD-fed mice
Tuzzerella	WD-fed mice
unknown Atopobiaceae	WD-fed mice
unknown Desulfovibrionaceae	WD-fed mice

Abbreviations used: ND, normal diet; WD, Western diet.
